# Genotyping of Endocervical *Chlamydia trachomatis* Strains and Detection of Serological Markers of Acute and Chronic Inflammation in Their Host

**Published:** 2012-06-19

**Authors:** Behrouz Taheri Beni, Anahita Jenab, Rasoul Roghanian, Hossein Motamedi, Naser Golbang, Pouran Golbang, Javad Zaeimi Yazdi

**Affiliations:** 1 Department of Biology, Faculty of Sciences, Shahid Chamran University, Ahvaz, Iran; 2 Department of Biology, Faculty of Sciences, University of Isfahan, Isfahan, Iran; 3 Shahid Beheshti Hospital, Isfahan, Iran; 4 Department of Pathobiology, School of Medicine, Yazd University of Medical Sciences, Yazd, Iran

**Keywords:** *Chlamydia trachomatis*, Genital Infection, Genotype, PCR, RFLP, Immune Markers

## Abstract

**Background:**

*Chlamydia trachomatis (C. trachomatis)* is the most prevalent cause of bacterial sexually transmitted infections (STI) recognized throughout the world. The aim of this study is to determine different genotypes of genital *C. trachomatis* and the association between the serological markers of inflammation and genotypes of *C. trachomatis* in sexually active women (n=80) attending Shahid Beheshti Hospital in Isfahan, Iran.

**Materials and Methods:**

In this descriptive study, endocervical swabs were collected from 80 women. There were 17 endocervical samples that showed positivity for *C. trachomatis* by plasmid polymerase chain reaction (PCR) using KL1 and KL2 primers. The *omp1* gene was directly amplified in 17 plasmid PCR positive samples and was used to differentiate the clinical genotypes by *omp1* gene PCR-restriction fragment length polymorphism (PCR-RFLP). The levels of IgG and IgA specific to C. trachmatis and C-reactive protein (CRP) were evaluated.

**Results:**

Based on restriction-digestion patterns, four genotypes were identified. Genotypes E (35.3%) and F (35.3%) were the most prevalent, followed by D/Da (23.5%) and K (5.9%). There was no significant association between genotypes and the presence of IgG and CRP. Patients infected with genotype E showed a serological marker of chronic inflammation, i.e. IgA seropositivity, significantly more than patients infected with other genotypes (p=0.042).

**Conclusion:**

Nested PCR could increase the sensitivity of *omp1* amplification. Based on the presence of IgA, chronic C. trachomatis infections were observed more frequently among genotype E-infected patients in our population.

## Introduction

*Clamydia trachomatis (C. trachomatis)* is the most common bacterial cause of sexually transmitted infections (STI). The World Health Organization estimates that approximately 92 million new cases of genital chlamydial infection occur worldwide annually (1, 2). Because 50% of infections in men and 80% in women are asymptomatic, the actual number of cases seems to be more than the estimated number (1, 3). Currently, 19 human genotypes and numerous variants including A, B/ Ba, C, D/Da, E, F, G/Ga, H, I/Ia, J, K, L1, L2/L2a, and L3 have been identified using polyclonal and monoclonal antibodies against the major outer membrane protein (MOMP) (4, 5). MOMP is a predominant antigen, which is different in various strains of *C. trachomatis* (6-8). Genotypes A-C (the trachoma biovars) cause conjunctivitis and lead to trachoma, the primary cause of preventable blindness in third world countries (8). Genotypes D-K are the most important cause of urogenital and neonatal infections. They are the most common cause of sexually transmitted genital infections, eliciting local acute epithelial infections, which can lead to pelvic inflammatory disease in women. Untreated infection or chronic infection can occasionally cause infertility, potentially fatal ectopic pregnancy, and premature delivery (7- 9). Genotypes L1-L3 cause the invasive disease known as lymphogranuloma venerum (7, 8).

Sequencing of the *omp1* gene in *C. trachomatis* showed a significant difference among genotypes (10). Restriction fragment length polymorphism (RFLP) analysis of *omp1* gene has been used to differentiate genotypes of *C. trachomatis* by using different enzymes (11-14).

Determination of the epidemiological relationship between the *C. trachomatis* genotype from different areas could be a suitable guideline for designing epidemiological programs for controlling chlamydial infections, and consequently controlling sexually transmitted diseases (STDs). Considering the importance of this issue, the aim of this study was to determine the prevalence of C. trachomatis genotypes in symptomatic cervical infections in Iranian women. A hypothesis currently under investigation states that genetic variations in the *C. trachomatis* genome may account for strain (genotype) differences in the course and outcome of infection with this bacteria (15-17). The chronic status in the course of a *C. trachomatis* infection is one of the most important aspects of this infection. It is associated with the persistence of the bacteria in the host cells that increases the risk of tubal factor subfertility (18). IgA antibodies are assumed to reflect chronic inflammation (19, 20). A level of C-reactive protein (CRP) >10 mg/l usually occurs in acute infections, and can be detected using common tests for CRP. A CRP level <1 mg/l indicates the absence of inflammation or infection (21). Because CRP is a general serological marker of acute inflammation, another object of this study is to detect the serological markers of acute and chronic infections in patients, with the intent to evaluate the differences among different genotypes.

## Materials and Methods

### Study population and sample collection

This descriptive study was approved by the Ethics Committee of Shahid Beheshti Hospital. Samples were collected after obtaining written informed consent from 80 patients who attended the Gynecology Outpatient Department of Shahid Beheshti Hospital in Isfahan, Iran in 2008. An endocervical swab from each individual was transferred to 5 ml of sterile phosphate buffered saline (PBS) and stored at -70˚C until DNA extraction. In addition, 5ml of peripheral blood was collected from each patient for serological investigation (22, 23).

### DNA extraction

The endocervical swab sample was removed from the vial and the PBS collection tube was centrifuged at 2000 rpm for 15 minutes. The supernatant was discarded and the bottom was vortexed and transferred to a 1.5 ml microtube. This step was followed by centrifugation at 2000 rpm for 15 minutes. The supernatant was then removed. Then, 400 μl of tris-base-EDTA (TE) solution that contained 10 mM tris–HCl, pH= 8.0 and 1 mM EDTA, was added. 4 μl proteinase K (10 μg/ml) and 4 μl triton 10% (v/v) were added and incubated at 55˚C for 90 minutes, followed by 95˚C for 30 minutes. These samples were maintained at -20˚C until used (22-24).

### Plasmid PCR

All 80 samples were examined by *C. trachomatis* plasmid-based PCR using KL1 and KL2 primers. Successful amplification of a 241 bp fragment of the bacterial endogenous plasmid genome was considered a positive result by polymerase chain reaction (PCR). The primers used for the *C. trachomatis* plasmid PCR were KL1 (5′-TCCGGAGCGAGTACGAAGA-3′) and KL2 (5′-AATCAATGCCCGGGATTGGT-3′; Metabion, Germany) (9, 22, 24). The final reaction mix contained 5 μl of the extracted DNA sample, 16 pM of each primers, 0.28 μM deoxynucleotide triphosphate (Cinnagen, Iran), 3 mM MgCl2 (Cinnagen, Iran), and 1 U of Taq polymerase (Cinnagen, Iran), for a total volume of 25 μl (9, 22, 24). The amplification protocol was 10 minutes of DNA denaturation at 94˚C followed by 40 cycles of amplification, with each cycle that consisted of denaturation at 94˚C for 1 minute, annealing at 55˚C for 1 minute, and extension at 72˚C for 1 minute. The final extension cycle at 72˚C was prolonged for 8 minutes (9, 22, 24). The PCR products were analyzed by 1.5% agarose gel electrophoresis. *C. trachomatis* genotype A was used as positive control and a sample that contained only distilled water was used as a negative control.

### Omp1 PCR

We examined 17 plasmid PCR-positive samples for *omp1* PCR. An approximately 1.2 kb fragment of the *omp1* gene was amplified in the 17 plasmid-based PCR positive samples using three primers: CT1 (forward strand: 5′- GCCGCTTTGAGTTCTGCTTCCTC- 3′), CT5 (reverse strand: 5′- ATTTACGTGAGCAGCTCTCTCAT-3′), and PCTM3 (forward strand: 5′- TCCTTGCAAGCTCTGCCTGTGGGGAATCCT- 3′; Gene Fanavaran) (14). Primary PCR was performed on 10 μl of the extracted DNA in a final reaction mixture of 50 μl. The final reaction mixture contained 10 mM tris-HCl (pH= 8.3; Cinnagen), 50 mM KCl (Cinnagen), 1.5 mM MgCl2 (Cinnagen), 200 μM from each deoxynucleoside triphosphate (dATP, dTTP, dGTP, and dCTP; Cinnagen), 25 ρmol of each primer CT1, CT5, and 1U of Taq DNA polymerase (Cinnagen). The amplification protocol was 5 minutes of DNA denaturation at 95˚C followed by 35 cycles of amplification, with each cycle that consisted of denaturation at 95˚C for 1 minute, annealing at 55˚C for 1 minute, and extension at 72˚C for 1.5 minutes. The final extension cycle at 72˚C was prolonged for 4 minutes (14). In this study, an Eppendorf Master Cycler Epgradient was used. The semi-nested PCR was carried out in the following manner: 1 μl of the primary PCR product (as the DNA template) was added to a prepared PCR mixture that contained primer PCTM3, located 22 bp downstream of CT1 and the previous primer, CT5. The position of CT1 is at 34-56 bp and PCTM3 is at 55-84 bp. The amplification conditions of the semi-nested PCR were the same as the conditions of the primary PCR. The PCR products were analyzed by 1% agarose gel electrophoresis (14). For a 1% agarose gel we added 1 g of agarose to 100 ml of 1x electrophoresis buffer and 0.5 μg/ml ethtidium bromide to the molten agarose. In this study, *C. trachomatis* genotype E was used as a positive control and a sample that contained distilled water instead of DNA was used as a negative control.

### RFLP

The *omp1* seminested PCR products were digested with restriction enzymes according to the previous study (12, 14). Restriction digestion was performed in two manners: i. single digestion with restriction enzyme *AluI*; ii. triple digestion with three enzymes *HpaII*, *EcoRI*, and *HinfI*. The first digestion was carried out with 10 μl of amplified DNA on 4 U of *AluI*, using the assay buffer recommended by the manufacturer, at 37˚C for 4 hours. The second digestion was performed on 10 μl of amplified DNA, first with 4 U of *HpaII* in 10 mM tris HCl (pH=7.6) and 10 mM MgCl2 at 37˚C for 4 hours. *HpaII* was then added in a 10 minute incubation at 60˚C. Next, 2 μl of 200 mM tris HCl (pH=8) and 75 mM NaCl were added and samples were incubated overnight with 4 U of *EcoRI* and Hinfl at 37˚C (12). Rodriguez et al. reported the single digestion differentiates between ten genotypes: A, C, E, F, G, I, J, K, L1, and L2, while B, Ba, D, H, and L3 show similar patterns. The triple digestion differentiates 11 genotypes: D, E, F, G, H, I, J, K, L1, L2, and L3 but genotypes A, C and B, Ba have similar patterns (12,14). In our study, a triple digestion was carried out to discriminate genotypes D/Da from B/Ba. Genotype D was not differentiated from Da, because D and Da have similar patterns in both single and triple digestion (12). CfoI digestion can be used to differentiate genotype D from Da (25).

Digestion products were analyzed by a 6% polyacrylamide gel stained with 15 μg of ethidium bromide per ml. For identification of the clinical strains, the RFLP pattern of each sample was compared with the *omp1* restriction fragment sizes (larger than 100 bp) presented in the previous study (12).

### Serological tests

Blood samples were taken from 80 women and the sera were used to determine the level of IgG and IgA antibodies against *C. trachomatis* using p-ELISA kits (Medac, Germany). The p-ELISA was based on a synthetic peptide from the immunodominant region of the major outer membrane protein. The ELISA kit used in this study was very specific for detecting *C. trachomatis* without any cross activity with other species of Chlamydia. The level of CRP was determined by using a CRP kit (Omega, UK). The sensitivity of the CRP test was 6 mg/L. Both tests were performed according to the manufacturers’ instructions.

### Statistical analysis

Analysis of the association of genotypes and the serological markers was carried out by a chisquare test. Data analysis was performed with SPSS statistical software version 15.0. P value less than 0.05 was considered significant.

## Results

**Fig 1:**
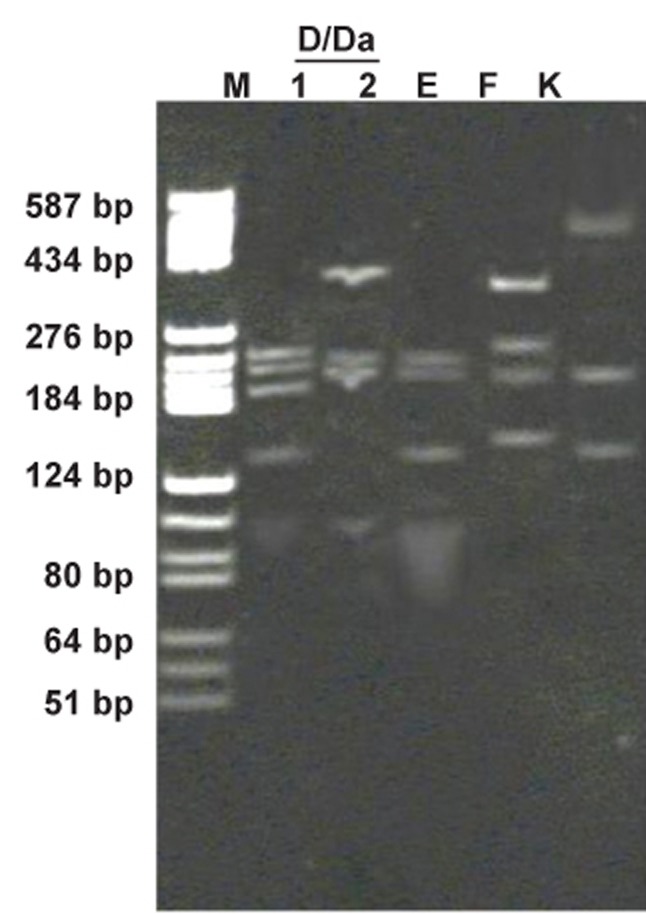
Restriction patterns were compared with acquired restriction patterns in reference 12. RFLP patterns of four clinical genital *C. trachomatis* strains (D/Da, E, F, and K) obtained by 6% polyacrylamide gel electrophoresis after restriction hydrolysis of the *omp1* gene-PCR products. Lane 1: *AluI* digestion of genotype D/Da. Lane 2: *HpaII-EcoRI-HinfI* digestion of genotype D/Da. Lanes E, F, and K indicate *AluI* digestion of genotypes E, F, and K, respectively. Lane M: pBR322 digested with HaeIII as the DNA size marker.

The successful amplification of a 241 bp fragment of the *C. trachomatis* plasmid genome was considered a positive result by plasmid-PCR. A total of 17 out of 80 samples (21.25%) were positive for *C. trachomatis* by KL1 and KL2 primers. *omp1* was successfully amplified by CT1-CT5 PCR (primary-PCR) in 14 of 17 samples (82.3%). In the three remaining samples, *omp1* was amplified after nested-PCR with PCTM3 and CT5 primers. *AluI* digestion resulted in four genotypes: D/ Da (or B/Ba), E, F and K. Digestion with *HpaII*, *EcoRI*, and *HinfI* were performed for distinguishing the D/Da and B/Ba genotypes from each other. The triple digestion patterns of these samples corresponded with genotype D/Da (Fig 1). Restriction patterns were compared with acquired restriction patterns in reference 12. Of 17 samples, there were 4 (23.5%) genotype D/Da, 6 (35.3%) genotype E, 6 (35.3%) genotype F, and 1 (5.9%) genotype K. RFLP patterns of *omp1*-seminested PCR products of four genotypes in single digestion and of one genotype (D/ Da) in triple digestion manner are shown in figure 1. Out of 17 patients, 5 (29.4%) showed positivity for IgG, 3 (17.6%) for IgA, and 6 (35%) for CRP. Table 1 shows the relationship between the serological characteristics of infections and genotypes.

**Table 1:** Serological features of infections in relation to C. trachomatis genotypes


Serological feature	Genotype D/Da	Genotype E	Genotype F	Genotype K	P value

**IgG**	1/4 (25%)	2/6 (33.3%)	2/6 (333%)	0(%)	0.42
**IgA**	0/4 (0%)	3/6 (50%)	0 (0%)	0(0%)	0.04
**CRP^a^**	2/4 (50%)	1/6 (16.7%)	3/6 (50%)	1/1 (100%)	0.18
**Without immunological**	1	2^b^	1	0	
**markers**					
**Total**	4	6	6	1	


a; C-reactive protein. b; Two samples of genotype E were IgG and IgA double-seropositive. Out of 6 genotype E samples, 2 samples were without immunological markers.

## Discussion

*C. trachomatis* is a common sexually transmitted infection with significant impact on public health. Therefore, effective epidemiological control as well as a correct and sensitive diagnostic method for *C. trachomatis* are required (22, 23). In order to develop epidemiological data and to detect various genotype infections, accurate and specific typing of *C. trachomatis* genotypes is necessary (12). Molecular techniques, like PCR-RFLP, are more sensitive and less time-consuming than immunofluorescence (12). Also, since immunotyping has serious limitations, a suitable and convenient tool that can be used to type and survey epidemiological studies is PCR-RFLP (12). In the current study, 17 out of 80 samples were positive with plasmid PCR. The plasmid-PCR showed 10x higher sensitivity than MOMP-PCR due to having ten copies of plasmid in the elementary body compared to the MOMP gene, which has only one (11, 24). Thus in this study plasmid-PCR was used as a gold standard.

Results of *omp1* PCR showed that the nested PCR could increase the sensitivity of *omp1* amplification, because the *omp1* fragment was amplified in 14 out of 17 samples using primary PCR with CT1 and CT5 primers (82.3%), whereas *omp1* was successfully amplified in the 3 remaining samples by using nested PCR with pCTM3 and CT5. This manner could be useful, particularly for direct PCR on crude suspensions of samples that contain low copy numbers of Chlamydia. RFLP patterns with fragments larger than 100 bp produced from the CT1-CT5 sequence of the *omp1* gene for the 15 C. trachomatis genotypes in the previous study were considered as reference patterns in this study (12), but for optimizing *omp1*-PCR in the present study, the pCTM3- CT5 sequence was amplified and used for RFLP. Based on BLAST searching at the NCBI, the first restriction sites for *AluI* (AGCT) is located at 63-66 bp of the *omp1* gene, and for *HinfI* (GAATC) at 78-82 bp of *omp1* gene, and both are apparent on the pCTM3 primer. Therefore, RFLP patterns of products of CT1- CT5-PCR and PCTM3-CT5-PCR in single digestion are different in the 64 bp fragment and in triple digestion in a 78 bp fragme nt, which both are smaller than 100 bp.

In this study, genotypes E and F were the most prevalent genotypes, followed by genotype D/Da and K. The first investigation on genotyping of *C. trachomatis* was performed in Ahvaz, Iran. This study showed that the most prevalent genotype was E (31.5 %), followed by F (23.1 %), D/Da (13 %), K (9.2 %), I (8.3 %), G (7.5 %), H (5.5 %), and J (1.9 %) (14). In other parts of the world, genotypes E, F, and D were responsible for the most genital *C. trachomatis* infections (26-29).

The acute phase protein CRP is a general serological marker of inflammation. In this study, there was no significant association between the level of CRP, IgG antibodies, and genotypes (p=0.18 and 0.42, respectively). Serum IgG antibodies against microorganisms usually remain detectable for many years, even after antibiotic treatment (30).

Detecting merely IgA antibody by p-ELISA, indicating that *C. trachomatis* infection may be present at an early stage of acute infection (31). The presence of high titer of IgA antibodies is associated with chronic inflammation (19, 20). In this study, we have shown that genotype E samples were significantly related to IgA seropositivity (p=0.042), which indicated chronic *C. trachomatis* infections were observed more frequently among genotype E. Molano et al. reported that chronic *C. trachomatis* infections were observed more frequently among D and E genotypes, with a lower frequency among genotypes B, H, I, and K (27). These researchers have also shown that in a mouse model, the duration of lower genital tract infection was longest with D and E genotypes, and improvement in the upper genital tract occurred more often in mice infected with D genotype compared to mice infected with H genotype (27).

Our data and the above mentioned studies have indicated that the course of a *C. trachomatis* infection (such as whether the infection will be cleared or chronic) may be influenced by differences among various genotypes. However, there have not been enough studies undertaken in these areas to prove this theory. Serological responses to various genotypes might differ in different areas and populations. They can be justified with the role of genetic variations in immunologically important host genes in the course and outcome of infectious. To our knowledge, the evaluation of the relationship between *C. trachomatis* genotypes and features of the serological response has not been carried out in Iran. Further studies need to be done on larger populations in different parts of the world.

## Conclusion

Our data showed that the nested PCR could increase the sensitivity of *omp1* amplification. Genotypes E and F were the most prevalent genotypes in this cohort. It was also shown that there was no significant association between the levels of CRP, IgG antibodies, and different genotypes. Furthermore, genotype E samples were significantly related to IgA seropositivity.
